# 3-(Dihy­droxy­bor­yl)anilinium 6-carb­oxy­pyridine-2-carboxyl­ate

**DOI:** 10.1107/S1600536812031790

**Published:** 2012-07-25

**Authors:** Chunhua Ge, Xiangdong Zhang, Rui Zhang, Chenglong Zhang

**Affiliations:** a College of Chemistry, Liaoning University, Shenyang, Liaoning 110036, People’s Republic of China

## Abstract

In the anion of the title molecular salt, C_6_H_9_BNO_2_^+^·C_7_H_4_NO_4_^−^, the dihedral angles between the –COO^2−^ and –CO_2_H groups and their attached ring are 4.02 (13) and 21.41 (10)°, respectively. The B atom in the cation adopts a *syn*–*syn* geometry and the dihedral angle between the –B(OH)_2_ group and its attached ring is 11.06 (5)°. In the crystal, O—H⋯O, N—H⋯O and N—H⋯N hydrogen bonds link the components into a three-dimensional network.

## Related literature

For general background, see: Hall (2005 ▶). For related structures, see: Li *et al.* (1995 ▶); SeethaLekshmi & Pedireddi (2006 ▶); Sokolov & MacGillivray (2006 ▶); Vega *et al.* (2010 ▶).
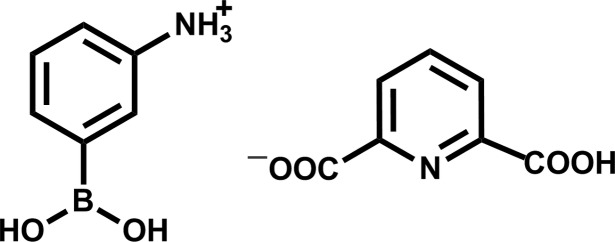


## Experimental

### 

#### Crystal data

C_6_H_9_BNO_2_^+^·C_7_H_4_NO_4_^−^
*M* *_r_* = 304.06
Monoclinic, 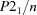

*a* = 7.7065 (6) Å
*b* = 14.0473 (10) Å
*c* = 13.0852 (10) Å
β = 106.963 (1)°
*V* = 1354.92 (18) Å^3^
*Z* = 4
Mo *K*α radiation
μ = 0.12 mm^−1^
*T* = 293 K
0.28 × 0.25 × 0.20 mm

#### Data collection

Bruker SMART CCD area-detector diffractometer
Absorption correction: multi-scan (*SADABS*; Bruker, 2001 ▶) *T*_min_ = 0.958, *T*_max_ = 0.989
8330 measured reflections
2677 independent reflections
2260 reflections with *I* > 2σ(*I*)
*R*_int_ = 0.018

#### Refinement

*R*[*F* ^2^ > 2σ(*F* ^2^)] = 0.037
*wR*(*F* ^2^) = 0.102
*S* = 1.05
2677 reflections
206 parameters
1 restraint
H atoms treated by a mixture of independent and constrained refinement
Δρ_max_ = 0.25 e Å^−3^
Δρ_min_ = −0.20 e Å^−3^

### 

Data collection: *SMART* (Bruker, 2001 ▶); cell refinement: *SAINT* (Bruker, 2001 ▶); data reduction: *SAINT*; program(s) used to solve structure: *SHELXS97* (Sheldrick, 2008 ▶); program(s) used to refine structure: *SHELXL97* (Sheldrick, 2008 ▶); molecular graphics: *SHELXTL* (Sheldrick, 2008 ▶); software used to prepare material for publication: Mercury (Macrae *et al.*, 2006 ▶), *PLATON* (Spek, 2009 ▶), *SHELXL97* and *WinGX* (Farrugia, 1999 ▶).

## Supplementary Material

Crystal structure: contains datablock(s) I, global. DOI: 10.1107/S1600536812031790/cv5313sup1.cif

Structure factors: contains datablock(s) I. DOI: 10.1107/S1600536812031790/cv5313Isup2.hkl

Supplementary material file. DOI: 10.1107/S1600536812031790/cv5313Isup3.cml

Additional supplementary materials:  crystallographic information; 3D view; checkCIF report

## Figures and Tables

**Table 1:** Hydrogen-bond geometry (Å, °)

*D*—H⋯*A*	*D*—H	H⋯*A*	*D*⋯*A*	*D*—H⋯*A*
O1—H1*A*⋯O3^i^	0.96 (2)	1.47 (3)	2.429 (2)	173 (2)
N2—H2*A*⋯O1^i^	0.89	2.42	2.808 (2)	107
N2—H2*A*⋯O3^i^	0.89	2.42	2.835 (2)	109
N2—H2*A*⋯N1^i^	0.89	2.08	2.955 (2)	169
N2—H2*B*⋯O3^i^	0.89	2.49	2.835 (2)	104
N2—H2*B*⋯O2	0.89	2.03	2.907 (2)	170
N2—H2*C*⋯O6^ii^	0.89	2.15	2.947 (2)	149
O5—H5⋯O2^iii^	0.82	2.04	2.712 (2)	139
O6—H6⋯O4^iv^	0.82	1.91	2.694 (2)	158

Symmetry codes: (i) 
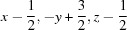
; (ii) 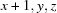; (iii) 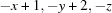; (iv) 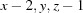.

**Fig. 1.**
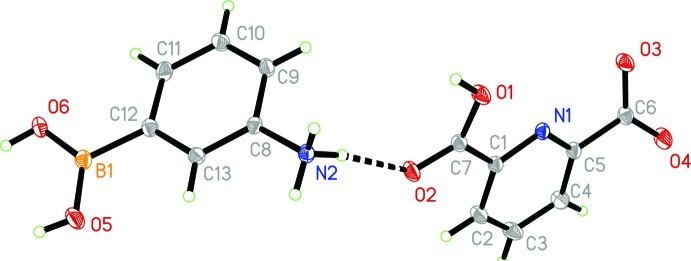
The molecular structure of the title compound, with displacement ellipsoids drawn at the 30% probability level.

## supplementary crystallographic information

### Comment

Boronic acid and its derivates have attracted great interest in various areas of materials science, catalysis, surface chemistry, organic synthesis, biochemistry, and luminosity (Hall, 2005). Boronic acid has been utilized to controucted covalent macrocycles compounds and organic frameworks as building block. Intermolecular interactions of boronic acid have now been well explored in the rapid development of organic supramolecular assemblies. A large variety of boronic acids have shown the application as new building blocks in crystal engineering through hydrogen-bonding interactions. 4-Carboxyphenylboronic acid was shown to produce second-sphere coordination networks with transition metals (SeethaLekshmi & Pedireddi, 2006). Cocrystallization of *trans*-1,2-bis(4-pyridyl)ethylene with phenylboronic acid could generate one-dimensional hydrogen bonded infinite ladder (Sokolov & MacGillivray, 2006). In the crystal of 3-aminophenyl boronic acid hydrochloride, each chloride ion is connected four organic ions by N—H···Cl and O—H···Cl hydrogen bonds (Li *et al.*, 1995). Bis[3-(dihydroxyboryl)anilinium] sulfate can provide a complex three-dimensional supramolecular network by hydrogen bonds (Vega *et al.*, 2010).

Here, we present the title compound - an organic salt of 3-(dihydroxyboryl)anilinium and 6-carboxypyridine-2-carboxylate (Fig. 1). In the crystal, intermolecular O—H···O, N—H···O and N—H···N interactions (Table 1) generate hydrogen-bonding network, which link cations and anions into three-dimensional structure.

### Experimental

An ethanolic solution of 2,6-pyridinedicarboxylic acid(0.5 mmol in 10 ml e thanol)was added dropwise to 3-aminophenyl boronic acid monohydrate (0.5 mmol in 5 ml e thanol) with stirring. Single crystals suitable for X-ray analysis were obtained by slow evaporation of the solvent at room temperature.

### Refinement

Atom H1A was located in a difference Fourier map and refined with a distance restraint O—H = 0.96 (2) Å. All other H atoms were positioned geometrically and refined using riding model, with C—H = 0.93 Å, O—H = 0.82 Å, N—H = 0.89 Å, and with *U*_iso_(H) = 1.2 *U*_eq_(C) or 1.5 *U*_eq_(O).

### Crystal data

**Table:**

C_6_H_9_BNO_2_^+^·C_7_H_4_NO_4_^−^	*F*(000) = 632
*M**_r_* = 304.06	*D*_x_ = 1.491 Mg m^−^^3^
Monoclinic, *P*2_1_/*n*	Mo *K*α radiation, λ = 0.71073 Å
Hall symbol: -P2yn	Cell parameters from 58 reflections
*a* = 7.7065 (6) Å	θ = 2.3–22.7°
*b* = 14.0473 (10) Å	µ = 0.12 mm^−^^1^
*c* = 13.0852 (10) Å	*T* = 293 K
β = 106.963 (1)°	Block, colourless
*V* = 1354.92 (18) Å^3^	0.28 × 0.25 × 0.20 mm
*Z* = 4	

### Data collection

**Table:**

Bruker SMART CCD area-detector diffractometer	2677 independent reflections
Radiation source: fine-focus sealed tube	2260 reflections with *I* > 2σ(*I*)
Graphite monochromator	*R*_int_ = 0.018
φ and ω scans	θ_max_ = 26.0°, θ_min_ = 2.2°
Absorption correction: multi-scan (*SADABS*; Bruker, 2001)	*h* = −9→8
*T*_min_ = 0.958, *T*_max_ = 0.989	*k* = −17→17
8330 measured reflections	*l* = −16→13

### Refinement

**Table:**

Refinement on *F*^2^	Primary atom site location: structure-invariant direct methods
Least-squares matrix: full	Secondary atom site location: difference Fourier map
*R*[*F*^2^ > 2σ(*F*^2^)] = 0.037	Hydrogen site location: inferred from neighbouring sites
*wR*(*F*^2^) = 0.102	H atoms treated by a mixture of independent and constrained refinement
*S* = 1.05	*w* = 1/[σ^2^(*F*_o_^2^) + (0.0541*P*)^2^ + 0.416*P*] where *P* = (*F*_o_^2^ + 2*F*_c_^2^)/3
2677 reflections	(Δ/σ)_max_ < 0.001
206 parameters	Δρ_max_ = 0.25 e Å^−^^3^
1 restraint	Δρ_min_ = −0.20 e Å^−^^3^

### Special details

**Table:**

Geometry. All e.s.d.'s (except the e.s.d. in the dihedral angle between two l.s. planes) are estimated using the full covariance matrix. The cell e.s.d.'s are taken into account individually in the estimation of e.s.d.'s in distances, angles and torsion angles; correlations between e.s.d.'s in cell parameters are only used when they are defined by crystal symmetry. An approximate (isotropic) treatment of cell e.s.d.'s is used for estimating e.s.d.'s involving l.s. planes.
Refinement. Refinement of *F*^2^ against ALL reflections. The weighted *R*-factor *wR* and goodness of fit *S* are based on *F*^2^, conventional *R*-factors *R* are based on *F*, with *F* set to zero for negative *F*^2^. The threshold expression of *F*^2^ > σ(*F*^2^) is used only for calculating *R*-factors(gt) *etc*. and is not relevant to the choice of reflections for refinement. *R*-factors based on *F*^2^ are statistically about twice as large as those based on *F*, and *R*- factors based on ALL data will be even larger.

### Fractional atomic coordinates and isotropic or equivalent isotropic displacement parameters (Å^2^)

**Table:**

	*x*	*y*	*z*	*U*_iso_*/*U*_eq_	
O2	0.99515 (15)	0.89532 (8)	0.27933 (8)	0.0301 (3)	
B1	0.1026 (2)	0.91402 (12)	−0.06619 (13)	0.0257 (4)	
C1	1.19186 (19)	0.93213 (10)	0.45205 (11)	0.0227 (3)	
C2	1.1728 (2)	1.02978 (11)	0.43673 (12)	0.0300 (4)	
H2	1.0986	1.0544	0.3729	0.036*	
C3	1.2659 (2)	1.08965 (11)	0.51780 (14)	0.0363 (4)	
H3	1.2534	1.1553	0.5101	0.044*	
C4	1.3776 (2)	1.05063 (11)	0.61038 (13)	0.0319 (4)	
H4	1.4459	1.0894	0.6650	0.038*	
C5	1.38602 (19)	0.95208 (10)	0.62036 (12)	0.0248 (3)	
C6	1.5061 (2)	0.90725 (10)	0.72088 (12)	0.0266 (3)	
C7	1.09722 (19)	0.86419 (10)	0.36402 (11)	0.0233 (3)	
C8	0.57150 (19)	0.81556 (10)	0.09336 (11)	0.0212 (3)	
C9	0.5358 (2)	0.76762 (10)	0.17760 (12)	0.0246 (3)	
H9	0.6274	0.7350	0.2274	0.030*	
C10	0.3611 (2)	0.76933 (11)	0.18593 (12)	0.0280 (3)	
H10	0.3345	0.7388	0.2426	0.034*	
C11	0.2256 (2)	0.81672 (11)	0.10949 (12)	0.0273 (3)	
H11	0.1085	0.8168	0.1157	0.033*	
C12	0.2593 (2)	0.86417 (10)	0.02364 (12)	0.0241 (3)	
C13	0.43773 (19)	0.86366 (10)	0.01759 (11)	0.0230 (3)	
H13	0.4662	0.8958	−0.0376	0.028*	
H1A	1.070 (4)	0.7337 (19)	0.329 (2)	0.103 (10)*	
N1	1.29358 (16)	0.89300 (8)	0.54318 (9)	0.0222 (3)	
N2	0.75722 (16)	0.81145 (8)	0.08587 (10)	0.0230 (3)	
H2A	0.7832	0.7520	0.0719	0.034*	
H2B	0.8341	0.8302	0.1475	0.034*	
H2C	0.7673	0.8497	0.0337	0.034*	
O1	1.13249 (15)	0.77632 (8)	0.38493 (8)	0.0309 (3)	
O3	1.46409 (15)	0.82127 (8)	0.73569 (9)	0.0334 (3)	
O4	1.63185 (16)	0.95337 (9)	0.77863 (10)	0.0424 (3)	
O5	0.15502 (15)	0.96244 (9)	−0.14174 (9)	0.0371 (3)	
H5	0.0661	0.9872	−0.1837	0.056*	
O6	−0.07164 (14)	0.90249 (8)	−0.06323 (8)	0.0312 (3)	
H6	−0.1416	0.9245	−0.1178	0.047*	

### Atomic displacement parameters (Å^2^)

**Table:**

	*U*^11^	*U*^22^	*U*^33^	*U*^12^	*U*^13^	*U*^23^
O2	0.0322 (6)	0.0294 (6)	0.0216 (5)	0.0028 (4)	−0.0035 (5)	0.0030 (4)
B1	0.0264 (9)	0.0232 (8)	0.0244 (9)	0.0018 (7)	0.0023 (7)	−0.0019 (6)
C1	0.0208 (7)	0.0259 (7)	0.0204 (7)	−0.0005 (6)	0.0045 (6)	0.0007 (6)
C2	0.0350 (9)	0.0271 (8)	0.0235 (8)	0.0021 (6)	0.0016 (7)	0.0039 (6)
C3	0.0485 (10)	0.0207 (7)	0.0351 (9)	0.0004 (7)	0.0050 (8)	0.0015 (7)
C4	0.0376 (9)	0.0249 (8)	0.0277 (8)	−0.0041 (7)	0.0008 (7)	−0.0043 (6)
C5	0.0222 (7)	0.0256 (7)	0.0242 (8)	−0.0002 (6)	0.0030 (6)	−0.0014 (6)
C6	0.0263 (8)	0.0265 (8)	0.0231 (8)	0.0017 (6)	0.0014 (6)	−0.0030 (6)
C7	0.0227 (7)	0.0261 (7)	0.0203 (7)	0.0012 (6)	0.0050 (6)	0.0022 (6)
C8	0.0190 (7)	0.0210 (7)	0.0220 (7)	−0.0012 (5)	0.0033 (5)	−0.0029 (5)
C9	0.0237 (7)	0.0250 (7)	0.0216 (7)	0.0014 (6)	0.0012 (6)	0.0013 (6)
C10	0.0287 (8)	0.0318 (8)	0.0237 (8)	−0.0001 (6)	0.0080 (6)	0.0043 (6)
C11	0.0201 (7)	0.0328 (8)	0.0290 (8)	0.0016 (6)	0.0071 (6)	−0.0001 (6)
C12	0.0237 (8)	0.0217 (7)	0.0245 (8)	0.0009 (6)	0.0033 (6)	−0.0025 (6)
C13	0.0247 (8)	0.0225 (7)	0.0208 (7)	−0.0001 (6)	0.0051 (6)	0.0013 (5)
N1	0.0193 (6)	0.0241 (6)	0.0209 (6)	−0.0004 (5)	0.0024 (5)	−0.0007 (5)
N2	0.0203 (6)	0.0241 (6)	0.0223 (6)	0.0000 (5)	0.0026 (5)	−0.0001 (5)
O1	0.0382 (7)	0.0241 (5)	0.0228 (6)	0.0001 (5)	−0.0031 (5)	0.0002 (4)
O3	0.0369 (7)	0.0280 (6)	0.0261 (6)	−0.0019 (5)	−0.0052 (5)	0.0029 (4)
O4	0.0396 (7)	0.0342 (6)	0.0369 (7)	−0.0055 (5)	−0.0145 (5)	−0.0009 (5)
O5	0.0291 (6)	0.0446 (7)	0.0344 (7)	0.0090 (5)	0.0042 (5)	0.0160 (5)
O6	0.0234 (6)	0.0377 (6)	0.0270 (6)	0.0021 (5)	−0.0010 (4)	0.0063 (5)

### Geometric parameters (Å, º)

**Table:**

O2—C7	1.2367 (17)	C8—C13	1.381 (2)
B1—O5	1.355 (2)	C8—C9	1.386 (2)
B1—O6	1.365 (2)	C8—N2	1.4640 (18)
B1—C12	1.582 (2)	C9—C10	1.382 (2)
C1—N1	1.3391 (18)	C9—H9	0.9300
C1—C2	1.388 (2)	C10—C11	1.387 (2)
C1—C7	1.510 (2)	C10—H10	0.9300
C2—C3	1.380 (2)	C11—C12	1.394 (2)
C2—H2	0.9300	C11—H11	0.9300
C3—C4	1.379 (2)	C12—C13	1.400 (2)
C3—H3	0.9300	C13—H13	0.9300
C4—C5	1.390 (2)	N2—H2A	0.8900
C4—H4	0.9300	N2—H2B	0.8900
C5—N1	1.3403 (19)	N2—H2C	0.8900
C5—C6	1.507 (2)	O1—H1A	0.963 (18)
C6—O4	1.2260 (19)	O5—H5	0.8200
C6—O3	1.2796 (19)	O6—H6	0.8200
C7—O1	1.2763 (18)		
			
O5—B1—O6	125.84 (14)	C9—C8—N2	117.31 (12)
O5—B1—C12	116.05 (14)	C10—C9—C8	118.56 (14)
O6—B1—C12	118.08 (13)	C10—C9—H9	120.7
N1—C1—C2	122.97 (13)	C8—C9—H9	120.7
N1—C1—C7	116.54 (12)	C9—C10—C11	119.81 (14)
C2—C1—C7	120.48 (13)	C9—C10—H10	120.1
C3—C2—C1	118.82 (14)	C11—C10—H10	120.1
C3—C2—H2	120.6	C10—C11—C12	122.15 (14)
C1—C2—H2	120.6	C10—C11—H11	118.9
C4—C3—C2	118.99 (14)	C12—C11—H11	118.9
C4—C3—H3	120.5	C11—C12—C13	117.39 (13)
C2—C3—H3	120.5	C11—C12—B1	121.94 (13)
C3—C4—C5	118.59 (14)	C13—C12—B1	120.64 (13)
C3—C4—H4	120.7	C8—C13—C12	120.12 (13)
C5—C4—H4	120.7	C8—C13—H13	119.9
N1—C5—C4	123.08 (14)	C12—C13—H13	119.9
N1—C5—C6	117.05 (13)	C1—N1—C5	117.46 (12)
C4—C5—C6	119.86 (13)	C8—N2—H2A	109.5
O4—C6—O3	126.49 (14)	C8—N2—H2B	109.5
O4—C6—C5	119.39 (14)	H2A—N2—H2B	109.5
O3—C6—C5	114.12 (13)	C8—N2—H2C	109.5
O2—C7—O1	125.03 (14)	H2A—N2—H2C	109.5
O2—C7—C1	119.97 (13)	H2B—N2—H2C	109.5
O1—C7—C1	114.99 (12)	C7—O1—H1A	114.1 (18)
C13—C8—C9	121.94 (13)	B1—O5—H5	109.5
C13—C8—N2	120.73 (13)	B1—O6—H6	109.5

### Hydrogen-bond geometry (Å, º)

**Table:**

*D*—H···*A*	*D*—H	H···*A*	*D*···*A*	*D*—H···*A*
O1—H1*A*···O3^i^	0.96 (2)	1.47 (3)	2.429 (2)	173 (2)
N2—H2*A*···O1^i^	0.89	2.42	2.808 (2)	107
N2—H2*A*···O3^i^	0.89	2.42	2.835 (2)	109
N2—H2*A*···N1^i^	0.89	2.08	2.955 (2)	169
N2—H2*B*···O3^i^	0.89	2.49	2.835 (2)	104
N2—H2*B*···O2	0.89	2.03	2.907 (2)	170
N2—H2*C*···O6^ii^	0.89	2.15	2.947 (2)	149
O5—H5···O2^iii^	0.82	2.04	2.712 (2)	139
O6—H6···O4^iv^	0.82	1.91	2.694 (2)	158

Symmetry codes: (i) *x*−1/2, −*y*+3/2, *z*−1/2; (ii) *x*+1, *y*, *z*; (iii) −*x*+1, −*y*+2, −*z*; (iv) *x*−2, *y*, *z*−1.
